# Combination of artificial intelligence endoscopic diagnosis and Kimura‐Takemoto classification determined by endoscopic experts may effectively evaluate the stratification of gastric atrophy in post‐eradication status

**DOI:** 10.1002/deo2.70029

**Published:** 2024-11-12

**Authors:** Kumiko Kirita, Seiji Futagami, Ken Nakamura, Shuhei Agawa, Nobue Ueki, Kazutoshi Higuchi, Mayu Habiro, Rie Kawawa, Yusuke Kato, Tomohiro Tada, Katsuhiko Iwakiri

**Affiliations:** ^1^ Department of Gastroenterology Nippon Medical School Hospital Graduate School of Medicine Tokyo Japan; ^2^ AI Medical Service, Inc. Tokyo Japan

**Keywords:** artificial intelligence, atrophic gastritis, gastric cancer, *Helicobacter pylori*, Kyoto Classification

## Abstract

**Background:**

Since it is difficult for expert endoscopists to diagnose early gastric cancer in post‐eradication status, it may be critical to evaluate the stratification of high‐risk groups using the advance of gastric atrophy or intestinal metaplasia. We tried to determine whether the combination of endoscopic artificial intelligence (AI) diagnosis for the evaluation of gastric atrophy could be a useful tool in both pre‐ and post‐eradication status.

**Methods:**

270 *Helicobacter pylori*‐positive outpatients (Study I) were enrolled and Study II was planned to compare patients (*n* = 72) with pre‐eradication therapy with post‐eradication therapy. Assessment of endoscopic appearance was evaluated by the Kyoto classification and Kimura‐Takemoto classification. The trained neural network generated a continuous number between 0 and 1 for gastric atrophy.

**Results:**

There were significant associations between the severity of gastric atrophy determined by AI endoscopic diagnosis and not having a regular arrangement of collecting venules in angle, visibility of vascular pattern, and mucus using Kyoto classification in *H. pylori*‐positive gastritis. There were significant differences (*p* = 0.037 and *p* = 0.014) in the severity of gastric atrophy between the high‐risk group and low‐risk group based on the combination of Kimura‐Takemoto classification and endoscopic AI diagnosis in pre‐ and post‐eradication status. The area under the curve values of the severity of gastric atrophy (0.674) determined by the combination of Kimura‐Takemoto classification and gastric atrophy determined by AI diagnosis was higher than that determined by Kimura‐Takemoto classification alone in post‐eradication status.

**Conclusion:**

A combination of gastric atrophy determined by AI endoscopic diagnosis and Kimura‐Takemoto classification may be a useful tool for the prediction of high‐risk groups in post‐eradication status.

## INTRODUCTION

Previous studies have reported that artificial intelligence (AI) endoscopic diagnosis using deep learning in digestive tract endoscopy, especially colonoscopy.^1^, ^2^, ^3^ Various gastrointestinal diagnoses with AI using deep learning for gastric cancer^4^ and *Helicobacter pylori* infection status^5^ have been reported. Expert endoscopists evaluate precancerous lesions based on endoscopic features such as intestinal metaplasia and gastric atrophy. Considering the risk factors for the development of gastric cancer, gastric atrophy, intestinal metaplasia, and enlarged‐fold appearance have been reported.^6^, ^7^, ^8^ There were some data about the usefulness of AI endoscopic diagnosis against gastric atrophy as precancerous lesions. Considering that the number of cases after *H. pylori* eradication is currently increasing in Japan, it is critical issues to determine data about AI endoscopic diagnosis against gastric atrophy in post‐eradication status. In addition, the new Kyoto Global Consensus Meeting on *H. pylori* gastritis proposed that the risk of *H. pylori*‐infected gastritis should be determined based on the extent of gastric atrophy.^9^ The usefulness of risk evaluation according to the Kyoto Classification of Gastritis using endoscopic features has been studied.^10^

In this study, we first tried to investigate whether the severity of gastric atrophy determined by AI endoscopic diagnosis was associated with any items of modified Kyoto classification or Kimura‐Takemoto classification. Then, secondly, in a small group study, we tried to determine whether gastric atrophy determined by AI endoscopic diagnosis could be useful for the evaluation of the severity of gastric atrophy between pre‐eradication status and post‐eradication status. Since early gastric cancer found in post‐eradication status appears with indistinct forms, such as tiny and flattened lesions,^21^, ^22^ it is difficult for expert endoscopists to find it. In view of the importance of the evaluation of the stratification of gastric mucosa in post‐eradication status, it may be critical issues to evaluate gastric risk including gastric atrophy via a combination of AI endoscopic diagnosis in post‐eradication status. Thus, we compared the Kimura‐Takemoto classification alone with the combination of gastric atrophy determined by AI endoscopic diagnosis and Kimura‐Takemoto classification. In this study, we first tried to determine whether endoscopic AI diagnosis for the evaluation of the severity of gastric atrophy as a precancerous lesion could be a useful tool in post‐eradication status.

## METHODS

### Patients

**FIGURE 1:**
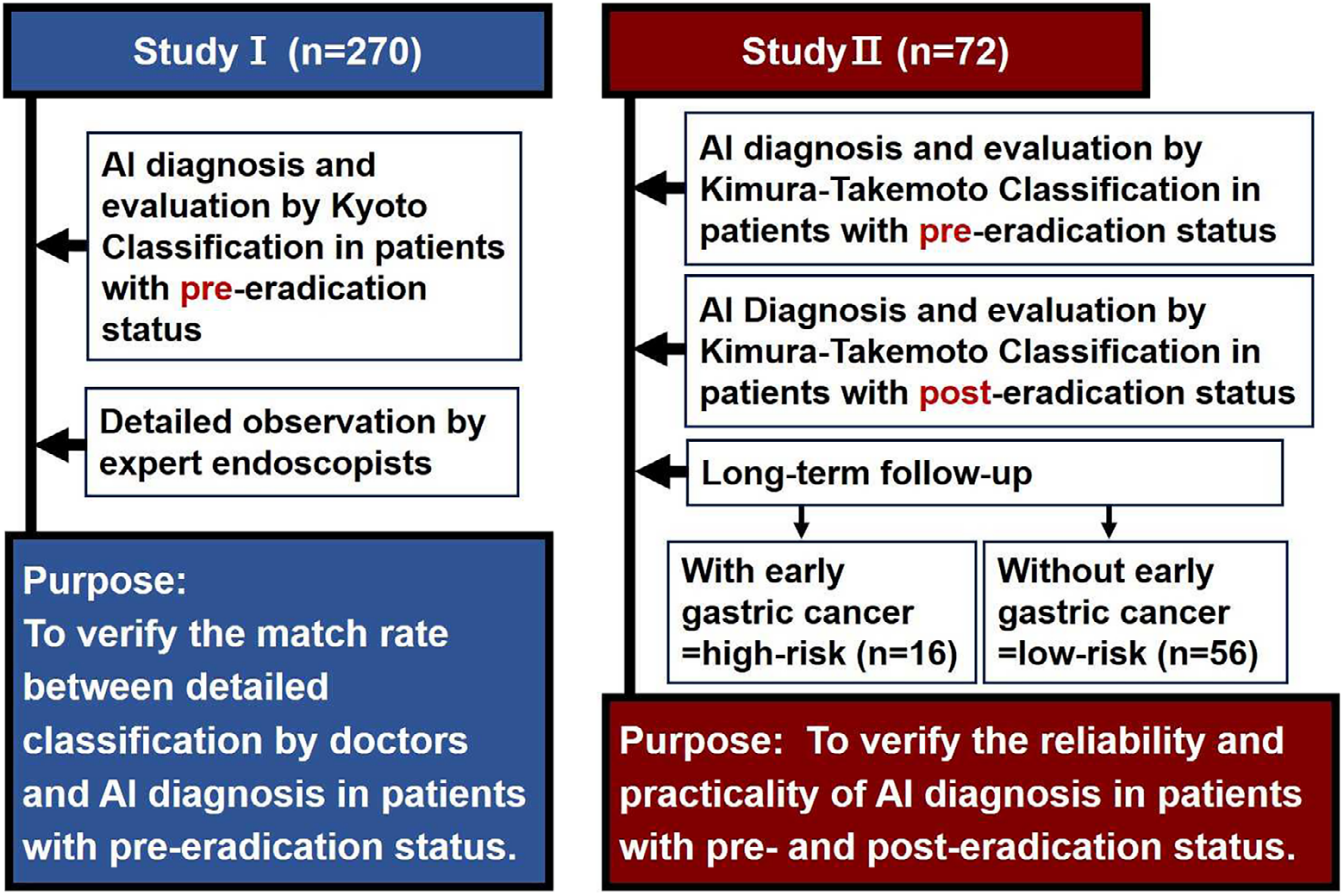
Study design.

This study enrolled 270 *H. pylori*‐positive outpatients, who either presented with upper gastrointestinal symptoms or received a periodical check‐up (Study I), and Study II was planned to compare the grade of gastric atrophy of patients (*n* = 72) with pre‐eradication therapy with three years after eradication therapy in Nippon Medical School Hospital during a period spanning from 2010 to 2021 (Figure 1). First, in study I, we classified 270 *H. pylori*‐positive gastritis using Kyoto classification and Kimura‐Takemoto classification, respectively. Then, the severity of gastric atrophy in 270 *H. pylori*‐positive gastritis was diagnosed using endoscopic AI. In Study I, we investigated which items of modified Kyoto classification were associated with gastric atrophy determined by endoscopic AI. We also investigated whether three items (regular arrangement of collecting venules [RAC] in angle, visibility of vascular pattern, and mucus) are actually linked to gastric atrophy determined by AI endoscopic diagnosis in *H. pylori*‐positive patients (*n* = 270). However, there were no significant relative associations between not having RAC in angle, visibility of vascular pattern, and mucus and gastric atrophy determined by AI endoscopic diagnosis (the area under the curve [AUC]: 0.564, 0.595, and 0.566, respectively; Figure S2). Since AI endoscopic diagnosis was not significantly associated with items of modified Kyoto classification such as not having RAC in angle, visibility of vascular pattern, and mucus in the view of AUC values (Study I), we tried to determine whether there were a significant relationship between gastric atrophy determined by AI endoscopic diagnosis and Kimura‐Takemoto classification in pre‐eradication status and post‐eradication status in Study II. In Study II, we divided 72 *H. pylori*‐positive patients into two groups, one is 16 patients with *H. pylori*‐positive patients a high‐risk group, developed into early gastric cancer in 6 years after eradication and the other is 56 *H. pylori*‐positive patients were a low‐risk group without early gastric cancer in the 6 years after eradication (Figure 1). In Study II, we tried to determine whether endoscopic AI diagnosis for the evaluation of the severity of gastric atrophy could be a useful tool in pre‐eradication and post‐eradication status. Exclusion criteria included renal failure and liver cirrhosis. Patients with previous gastroduodenal surgery and recent use of non‐steroidal anti‐inflammatory drugs (NSAIDs) at endoscopy were also excluded. *H. pylori* infection was determined by the^13^ C‐urea breath test, the positivity of *H. pylori* IgG antibody, or histological identification. Written informed consent was obtained from all subjects prior to undergoing upper gastrointestinal endoscopy. The study protocol was approved by the Ethics Review Committee of Nippon Medical School Hospital (490‐31‐19).

### Kimura‐Takemoto classification as reference standard

We compared the severity of gastric atrophy diagnosed by expert endoscopists using the Kimura‐Takemoto classification as a reference standard.^5^ Gastric atrophy was classified based on grades C‐0 (none), C‐I, C‐II, C‐III, O‐I, O‐II, and O‐III according to the endoscopic–atrophic‐border scale.

### Assessment of endoscopic appearance based on modified Kyoto classification

Endoscopic findings described in Kyoto classification of gastritis such as the presence or absence of RAC, enlarged gastric folds, nodularity, the existence of mucus, visible vascular pattern, diffuse and/or spotty redness, or patchy redness of the gastric mucosa were scored as follows: 0, absent; and 1, present for each patient based.^14^

### Training image sets

After selection, a total of 11,497 images, evaluated as the gold standard for the AI diagnosis, were collected for 4373 with gastric atrophy and 7124 without gastric atrophy as a training image data set determined by expert endoscopists in Osaka International Cancer Institute. The six classes are based on the presence or absence of atrophy for each region or view (antrum, body antegrade view, and body retroflex view) based on a modified previous study.^15^

### Test image sets and evaluation algorithm

A total of 7724 images from Study I and Study II were evaluated by the CNN from Nippon Medical School Hospital. The images were taken by various endoscopes (GIF‐H290Z [51/270], GIF‐H260Z [87/270], GIF‐H260 [96/270], and GIF‐XP260N [36/270] with VISERA ELITE system [Olympus]). The definition of gastric atrophy in test image sets was determined by AI based on the training image sets.

The endoscopic images are representatively shown in Figure S1. When expert endoscopists judged gastric atrophy in both views (Corpus‐down view and Corpus‐up view), it was classified as score 2. In addition, expert endoscopists judged no atrophy in the Corpus‐down and‐up view, and the atrophy in the cardia is classified as score 1. When expert endoscopists judged no atrophy in both views (Corpus‐down view and Corpus‐up view) and no atrophy in cardia, it was classified as score 0 (Figure S1). The gastroenterologists as expert endoscopists for this study had at least 10 years of experience performing endoscopies.

### Statistical analysis

Two‐tailed unpaired *t*‐test was performed to compare continuous variables, and Pearson's chi‐square test was performed to compare categorical variables. Receiver operating characteristic (ROC) curves were created by plotting sensitivity, as a proportion, versus (1‐specificity), as a proportion. All analyses were performed using SPSS (version 27.0; IBM Corp.), and *p*‐values <0.05 were considered to be statistically significant.

## RESULTS

### Clinical characteristics and endoscopic features in Kyoto classification in patients with *H. pylori*‐positive gastritis

In study I, we classified 270 *H. pylori*‐positive gastritis using the Kyoto classification and Kimura‐Takemoto classification, respectively. Clinical characteristics (age 59.5±13.9, gender: F/M: 138/132) in study I are exhibited in Table 1. In the Kimura‐Takemoto classification, in 270 *H. pylori*‐positive gastritis, the grade of gastric atrophy was the following: C‐I and C‐II: *n* = 54 (20%), C‐III: *n* = 19 (7%), and O‐I, O‐II, and O‐III: *n* = 197 (73%).

**TABLE 1:** Characteristics of the patients in each study.

	Study I (*n* = 270)		Study II (*n* = 72)		
	*H. pylori*‐positive gastritis	*p*‐value	High‐risk group (*n* = 16)	Low‐risk group (*n* = 56)	*p*‐value
Age (years)	59.5 ± 13.9		69.6 ± 12.1	63.2 ± 11.4	0.055
Gender (male/female)	132/138	0.970	15/1	30/26	0.003^*^

Data are presented as mean ± standard deviation.

Two‐tailed unpaired t‐test was performed to compare age, and Pearson's chi‐square test was performed to compare gender. All analyses were performed using SPSS (version 27.0; IBM Corp.).

Not having RAC in angle, visibility of vascular pattern and mucus were highly (92.6%, 63.3%, and 63.7%) confirmed in 270 *H. pylori*‐positive gastritis (Table 2, Study I).

**TABLE 2:** Kyoto Classification of patients by endoscopists in Study I.

Number of patients (percentage of total patients before eradication of *H. pylori*, *n* = 270)					
Diffuse redness	62 (23.0%)	Swelling of area gastrica	97 (35.9%)	Not having RAC in the angle	250 (92.6%)
Spotty redness	111 (41.1%)	Nodularity	25 (9.3%)	Fundic gland polyp	3 (1.1%)
Enlarged fold	47 (17.4%)	Kammrotung	24 (8.9%)	Flat erosion	24 (8.9%)
Atrophic fold	24 (8.9%)	Patchy redness	116 (43.0%)	Raised erosion	34 (12.6%)
Visibility of vascular pattern	171 (63.3%)	Xanthoma	45 (16.7%)	Bleeding erosion	9 (3.3%)
Mucosal swelling	154 (57.0%)	Mucus	172 (63.7%)	Hematin	34 (12.6%)

Abbreviation: RAC, regular arrangement of collecting venules.

### Which items in the Kyoto classification were associated with the severity of gastric atrophy by endoscopic AI

Since endoscopic AI highly diagnosed *H. pylori*‐positive gastritis patients (174/270: 64.4 %) as score 2, we investigated which items of modified Kyoto classification were associated with gastric atrophy determined by endoscopic AI. Although not having RAC in angle, visibility of vascular pattern, and mucus were higher in patients diagnosed with severe atrophy by AI (97.1%, 70.1%, and 68.4%) and significantly associated with AI scores (*p <* 0.001, *p* = 0.002, and *p* = 0.031; Table 3, Study I) in *H. pylori*‐positive gastritis, there were some discrepancies of frequencies among not having RAC in angle, visibility of vascular pattern, and mucus in patients diagnosed with severe atrophy by AI. As not having RAC in angle, visibility of vascular pattern, and mucus will seem to be associated with gastric atrophy determined by AI endoscopic diagnosis in severe gastric atrophy in *H. pylori*‐positive gastritis, we also investigated whether three items are actually linked to gastric atrophy determined by AI endoscopic diagnosis in *H. pylori*‐positive patients (*n* = 270) in Figure S2. However, there were no significant relative associations between not having RAC in angle, visibility of vascular pattern, and mucus and gastric atrophy determined by AI endoscopic diagnosis (AUC: 0.564, 0.595, and 0.566, respectively) in Figure S2.

**TABLE 3:** Kyoto Classification of patients diagnosed with severe atrophy by artificial intelligence (*n* = 174) in Study I.

Number of patients (percentage of total patients before eradication of *H. pylori*, *n* = 174)					
Diffuse redness	44 (25.3%)	Swelling of area gastrica	68 (39.1%)	Not having RAC in the angle	169 (97.1%)^*^
Spotty redness	77 (41.3%)	Nodularity	15 (8.6%)	Fundic gland polyp	2 (1.1%)
Enlarged fold	35 (20.1%)	Kammrotung	12 (6.9%)	Flat erosion	11 (6.3%)
Atrophic fold	20 (11.5%)	Patchy redness	78 (44.8%)	Raised erosion	22 (12.6%)
Visibility of vascular pattern	122 (70.1%)^*^	Xanthoma	33 (19.0%)	Bleeding erosion	4 (2.3%)
Mucosal swelling	98 (56.3%)	Mucus	119 (68.4%)^*^	Hematin	16 (9.2%)

Abbreviations: AI, artificial intelligence; RAC, regular arrangement of collecting venules.

Pearson's chi‐square test was performed to test whether each item was significantly related to the AI score.

All analyses were performed using SPSS (version 27.0; IBM Corp.).

* Items in which the percentage of patients was greater than 50% and the AI score was significantly related.

### Comparison of Kimura‐Takemoto classification with the severity of gastric atrophy determined by endoscopic AI between high‐risk group and low‐risk group

We compared the severity of gastric atrophy between the high‐risk group and the low‐risk group. In the pre‐eradication status, there were significant differences (*p* = 0.009) in the severity of gastric atrophy between the high‐risk group and low‐risk group using the Kimura‐Takemoto classification (Table 4, Study II). In contrast, in the post‐eradication status, there were no significant differences (*p* = 0.082) in the severity of gastric atrophy between the high‐risk group and the low‐risk group using the Kimura‐Takemoto classification (Table 4, Study II).

**TABLE 4:** Comparison of Kimura‐Takemoto Classification and the degree of gastric atrophy determined by artificial intelligence in Study II.

	High‐risk group		Low‐risk group		*p*‐value	
	Pre‐eradication	Post‐eradication	Pre‐eradication	Post‐eradication	Pre‐eradication	Post‐eradication
Kimura‐Takemoto Classification (C‐I, C‐II /C‐III/O‐I, O‐II, and O‐III)	1/0/15	1/2/13	14/13/29	12/16/28	0.009^*^	0.082
Cardia^†1^ (0/1)	7/9	3/13	28/28	26/30	0.659	0.047^*^
Antrum^†1^ (0/1)	5/11	1/15	17/39	11/45	0.945	0.205
Corpus‐up^†1^ (0/1)	9/7	2/14	25/31	13/43	0.412	0.352
Corpus‐down^†1^ (0/1)	7/9	0/16	22/34	16/40	0.748	0.015^*^
AI score (0/1/2)	5/0/11	0/0/16	16/0/40	8/1/47	0.835	0.230
Combination of Kimura‐Takemoto Classification and AI score ^†2^ (0/1)	5/11	3/13	34/22	30/26	0.037^*^	0.014^*^

Abbreviations: AI, artificial intelligence, Pre‐eradication: before the eradication of *H. pylori*, Post‐eradication: after the eradication of *H. pylori*.

Pearson's chi‐square test was performed to compare the groups.

All analyses were performed using SPSS (version 27.0; IBM Corp.).

^*^ In the pre‐eradication group, there were significant differences in the severity of gastric atrophy between the high‐and low‐risk group using Kimura‐Takemoto classification(*p* = 0.009). In the post‐edradication group, there were significant differences in the severity of gastric atrophy between the high‐and low‐risk group using endoscopic AI diagnosis in cardia and corpus‐down (*p* = 0.047 and *p* = 0.015). In addition, in both pre‐ and post‐eradication groups, there were significant differences in the severity of gastric atrophy between the high‐risk group and low‐risk group using combination of Kimura‐Takemoto classification and endoscopic AI diagnosis (*p* = 0.037 and *p* = 0.014).

^†1^ : 1; Kimura‐Takemoto Classification is O‐I, O‐II, O‐III, and the AI score of each part is moderate or high, 0; otherwise.

^†2^ : 1; Kimura‐Takemoto Classification is O‐I, O‐II, O‐III and AI score is 2, 0; otherwise.

Then, in the pre‐eradication status, there were no significant differences (*p* = 0.659, *p* = 0.945, and *p* = 0.412, *p* = 0.748) in the severity of gastric atrophy between the high‐risk group and low‐risk group using endoscopic AI diagnosis in the cardia, antrum, corpus‐up, and corpus‐down. Then, in the post‐eradication status, there were no significant differences (p = 0.205 and *p* = 0.352) in the severity of gastric atrophy between the high‐risk group and low‐risk group using endoscopic AI diagnosis in cardia and antrum. Interestingly, in the post‐eradication, there were significant differences (*p* = 0.047 and *p* = 0.015) in the severity of gastric atrophy between the high‐risk group and low‐risk group using endoscopic AI diagnosis in cardia and corpus‐down (Table 4, Study II). Therefore, in both pre‐and post‐eradication, there were significant differences (*p* = 0.037 and *p* = 0.014) in the severity of gastric atrophy between the high‐risk group and low‐risk group using a combination of Kimura‐Takemoto classification and endoscopic AI diagnosis (Table 4, Study II). In addition, since the ratio of open‐type atrophy (13/16) in the high‐risk group in post‐eradication status was significantly higher than that (28/56) in the low‐risk group, Kimura‐Takemoto classification alone using open‐type and closed‐type also was useful for the detection of the high‐risk group (Table 4).

### Comparison of AUC values of the severity of gastric atrophy determined by AI endoscopic diagnosis with by combination of AI and Kimura‐Takemoto classification

**FIGURE 2:**
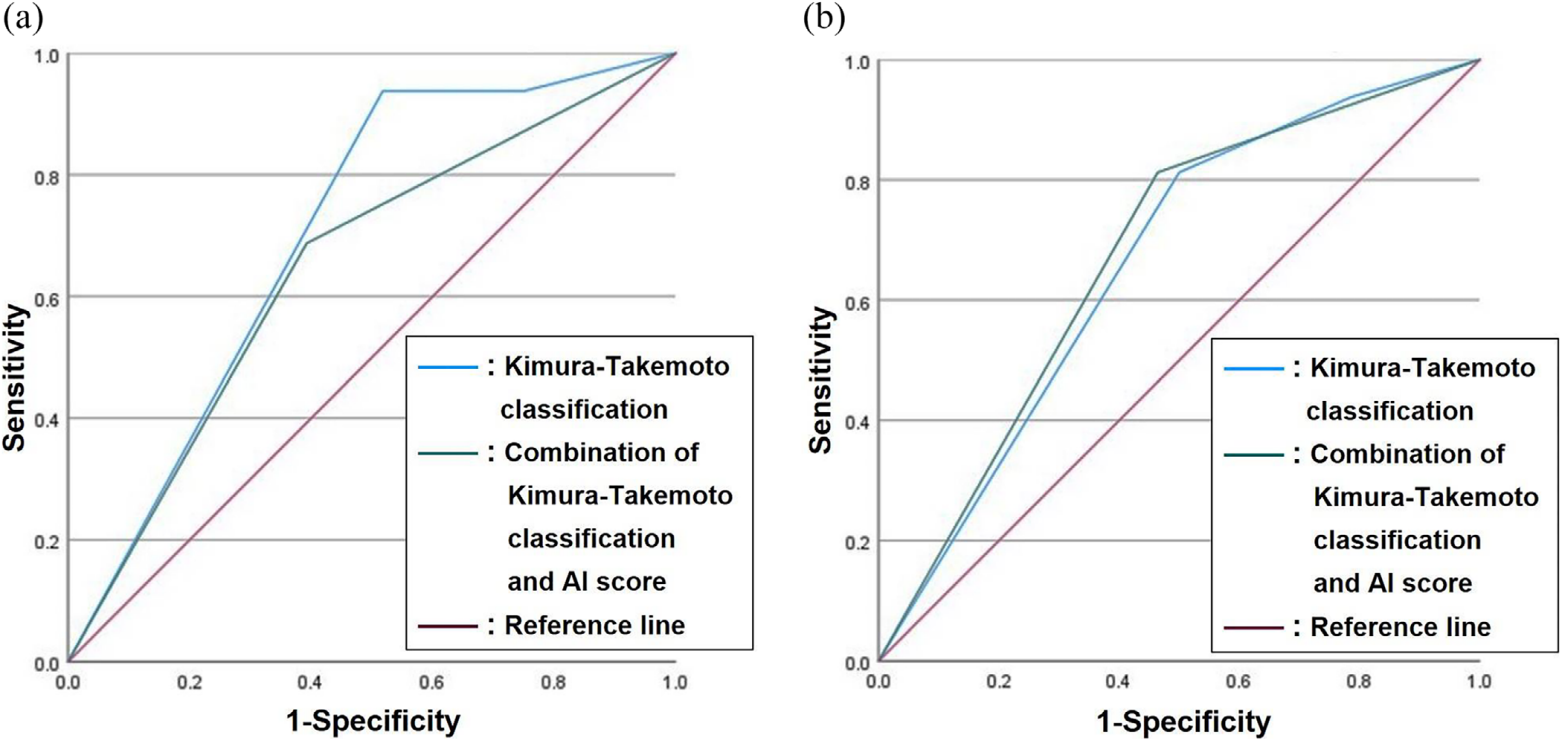
(a) Comparison of area under the curve (AUC) values in pre‐eradication status in Study II. Receiver operating characteristic (ROC) curves. The AUC values were 0.703 for Kimura‐Takemoto Classification ——, 0.647 for combination of Kimura‐Takemoto Classification and Artificial Intelligence (AI) score ——, Reference line ——. ROC curves were created by plotting sensitivity, as a proportion, versus (1‐specificity), as a proportion, using SPSS. The sensitivity/specificity of AUC data was following as the Kimura‐Takemoto classification alone in pre‐eradication status (0.938/0.482) and the combination of Kimura‐Takemoto classification and gastric atrophy determined by AI endoscopic diagnosis (0.688/0.607) in pre‐eradication status. (b) Comparison of area under the curve (AUC) values in post‐eradication status in Study II. Receiver operating characteristic (ROC) curves. The AUC values were 0.661 for Kimura‐Takemoto Classification ——, 0.674 for combination of Kimura‐Takemoto Classification and Artificial Intelligence (AI) score ——, Reference line ——. ROC curves were created by plotting sensitivity, as a proportion, versus (1‐specificity), as a proportion, using SPSS. The sensitivity/specificity of AUC data was following as the Kimura‐Takemoto classification alone in post‐eradication status (0.813/0.500), and the combination of Kimura‐Takemoto classification and gastric atrophy determined by AI endoscopic diagnosis in post‐eradication status (0.813/0.536).

To compare AUC values in the differences between the severity of gastric atrophy determined by the Kimura‐Takemoto classification alone and the severity of gastric atrophy determined by a combination of Kimura‐Takemoto classification and gastric atrophy determined by AI endoscopic diagnosis in the pre‐eradication status, we plotted ROC between two groups. The AUC value determined by the Kimura‐Takemoto classification was 0.703 and the AUC value determined by a combination of Kimura‐Takemoto classification and gastric atrophy determined by AI endoscopic diagnosis was 0.647 in the pre‐eradication status, respectively (Figure 2A, Study II). In contrast, the AUC value determined by the Kimura‐Takemoto classification was 0.661 and the AUC value determined by a combination of Kimura‐Takemoto classification and gastric atrophy determined by AI endoscopic diagnosis was 0.674 in the post‐eradication status, respectively (Figure 2B, Study II). Thus, the AUC value determined by a combination of Kimura‐Takemoto classification and gastric atrophy determined by AI endoscopic diagnosis was higher compared to the AUC value determined by Kimura‐Takemoto classification alone in post‐eradication status (Figure 2B, Study II). The AUC value for gastric atrophy determined by AI endoscopic diagnosis alone in pre‐eradication was 0.487 and the AUC value for gastric atrophy determined by AI endoscopic diagnosis alone in post‐eradication status was 0.580 (data not shown).

## DISCUSSION

The major findings of this study are: (1) in *H. pylori*‐positive gastritis, there were significant associations between the severity of gastric atrophy determined by AI endoscopic diagnosis and not having RAC in angle, visibility of vascular pattern, and mucus using modified Kyoto classification; (2) both pre‐ and post‐eradication status, there were significant differences in the severity of gastric atrophy between the high‐risk group and the low‐risk group based on the combination of Kimura‐Takemoto classification and endoscopic AI diagnosis; and (3) the AUC values were 0.661 determined by Kimura‐Takemoto classification, and 0.674 determined by the combination of Kimura‐Takemoto classification and gastric atrophy determined by AI endoscopic diagnosis in the post‐eradication status.

Although previous studies have reported that the AI endoscopic diagnosis system was a useful tool to detect high‐risk gastric mucosa or *H. pylori*‐positive gastric mucosa in pre‐eradication status,^16^, ^17^, ^18^ in the post‐eradication status, the combination of gastric atrophy determined by AI endoscopic diagnosis and Kimura‐Takemoto classification was more useful compared to Kimura‐Takemoto classification alone. Thus, the Kimura‐Takemoto classification alone also was useful for the detection of high‐risk groups in pre‐eradication status as described in Table 4. Since the severity of atrophic gastritis is well established as an indicator of increased risk for developing gastric cancer,^12^, ^13^, ^19^, ^20^ the evaluation of gastric atrophy determined by AI may be very important to predict the risk for advanced gastric cancer. In this study, we focused on the combination of gastric atrophy determined by AI endoscopic diagnosis and Kimura‐Takemoto classification to evaluate the severity of gastric atrophy more effectively compared to Kimura‐Takemoto classification alone in post‐eradication status.

Tao et al have also reported that the accuracy of the endoscopic AI system for mild gastric atrophy was higher than that for severe gastric atrophy.^16^ Since the number of cases after *H. pylori* eradication is currently increasing in Japan, further studies will be needed to clarify why the combination of gastric atrophy determined by AI endoscopic diagnosis and Kimura‐Takemoto classification could effectively evaluate the severity of gastric atrophy in the post‐eradication therapy as described in Figure 2. This tendency for gastric atrophy determined by AI endoscopic diagnosis has been reported to be a similar tendency to those in intestinal metaplasia.^16^ In addition, it may be difficult for expert endoscopists to diagnose the fainting of the border of the gastric atrophy through the re‐epithelialization and improvement of gastric atrophy after eradication.^11^ Thus, early gastric cancer in post‐eradication status increased and it is difficult for expert endoscopists to find it. In our data, gastric atrophy determined by AI endoscopic diagnosis in post‐eradication status may be additionally a useful tool for supporting expert endoscopists.

Although several studies have reported that gastric atrophy determined by AI endoscopic diagnosis was developed to find early gastric cancer in *H. pylori*‐positive patients as well as expert endoscopists,^21^, ^22^ in post‐eradication status, even expert endoscopists were difficult to find early gastric cancer. Even after the loss or eradication of *H. pylori* colonization, DNA methylation persists to some extent.^23^ Therefore, it may be critical issues to clarify whether gastric atrophy determined by AI endoscopic diagnosis could estimate the risk of gastric cancer in post‐eradication status. This study has several limitations. First, this was a retrospective study in two institutions. Second, we used only high‐quality endoscopic images for training images, but test images taken by various endoscopes including low‐quality endoscopes. Third, in this study, gastric atrophy determined by AI endoscopic diagnosis did not detect early gastric cancer directly. Fourth, in *H. pylori*‐positive gastritis, not having RAC in angle will be significantly associated with AI endoscopic diagnosis in severe gastric atrophy such as AI score 2. In contrast, not having RAC in angle will be relatively associated with AI endoscopic diagnosis in mild gastric atrophy such as AI score 0 in *H. pylori*‐positive gastritis. Considering the above issues, there were no significant relative associations between not having RAC in angle and gastric atrophy determined by AI endoscopic diagnosis (AUC: 0.564; Figure S2).

In addition, further studies will be needed to investigate whether AI endoscopic diagnosis for intestinal metaplasia, enlarged fold, nodularity, and diffuse redness could be associated with high‐risk groups, respectively. Taken together, in this study we addressed that gastric atrophy determined by AI endoscopic diagnosis may be useful to estimate mild gastric atrophy compared to expert endoscopists and especially, in post‐eradication status, the combination of AI endoscopic supports system can effectively evaluate the stratification of gastric atrophy as high‐risk factor.

## CONFLICT OF INTEREST STATEMENT

None.

## ETHICS STATEMENT

This study has been approved by the Research Ethics Review Committee of Nippon Medical School Hospital (Approval number: 490‐31‐19).

## PATIENT CONSENT STATEMENT

Obtained from all patients prior to undergoing upper gastrointestinal endoscopy

## Supporting information



Figure S1: Artificial Intelligence (AI) score and typical images. A: typical endoscopic images for AI diagnosis, a: moderate to severe, b: mild to moderate, c: none to mild. B: The degree of gastric atrophy determined by AI.

Figure S2: Comparison of area under the curve (AUC) values in H. pylori‐positive patients in Study I. Receiver operating characteristic (ROC) curves. The AUC values were 0.595 for visibility of vascular pattern —, 0.564 for not having regular arrangement of collecting venules (RAC) in angle —, 0.566 for mucus —, Reference line —.
