# Supplementary figures and images for: Combination of artificial intelligence endoscopic diagnosis and Kimura‐Takemoto classification determined by endoscopic experts may effectively evaluate the stratification of gastric atrophy in post‐eradication status
Source: DEN Open. 2024 Nov 12;5(1):e70029. doi: 10.1002/deo2.70029 (PMC11555298; doi:10.1002/deo2.70029)

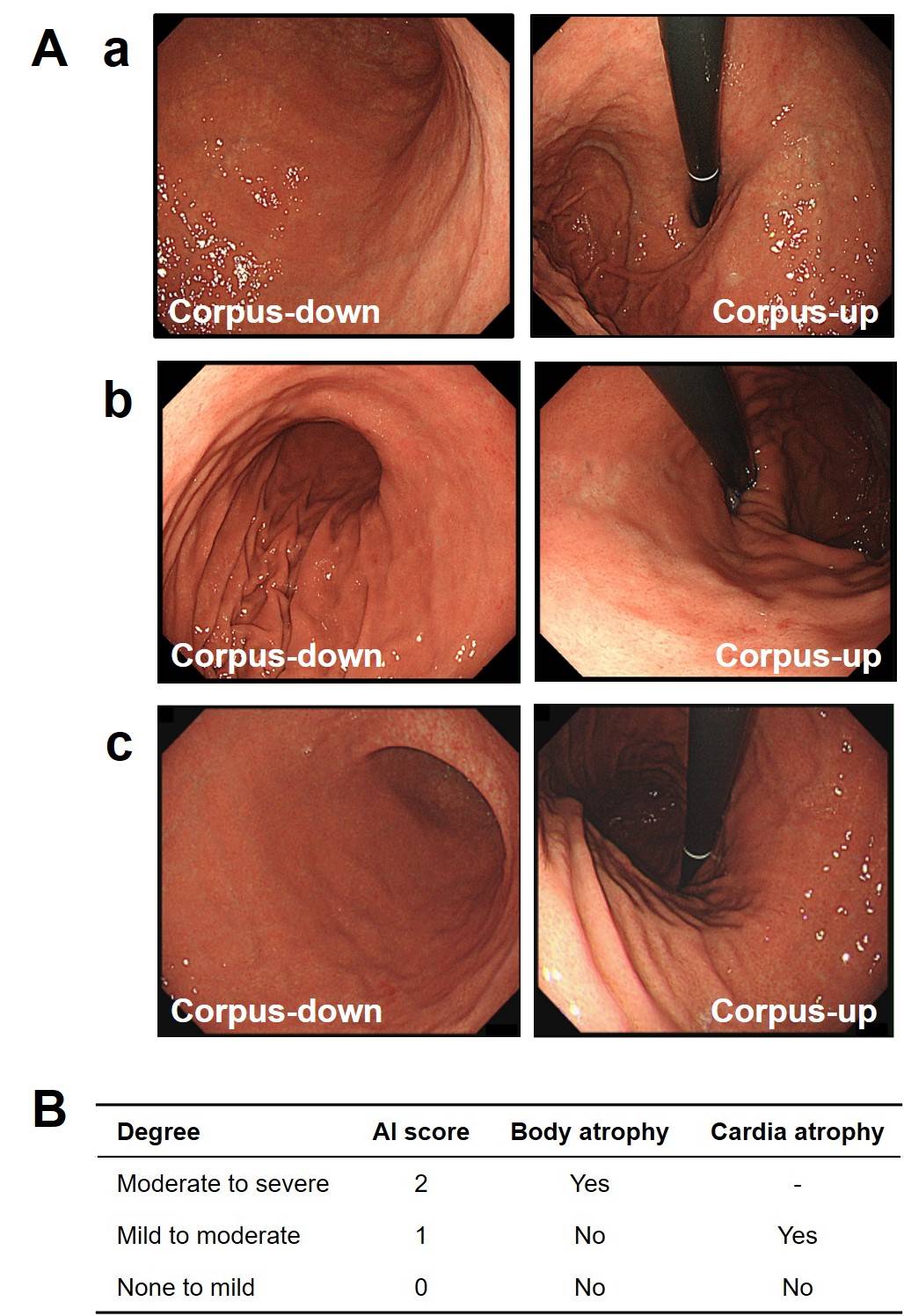

Supplement: Supplementary file 1 — Figure S1: Artificial Intelligence (AI) score and typical images. A: typical endoscopic images for AI diagnosis, a: moderate to severe, b: mild to moderate, c: none to mild. B: The degree of gastric atrophy determined by AI. [file DEO2-5-e70029-s002.jpg]

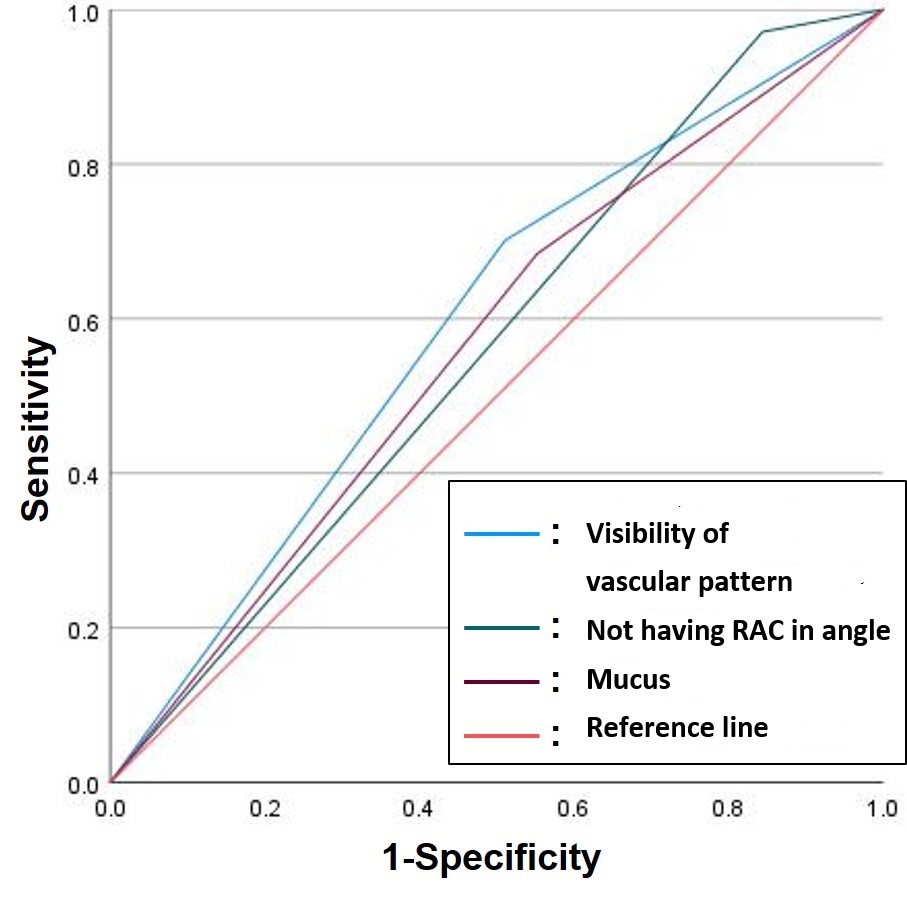

Supplement: Supplementary file 2 — Figure S2: Comparison of area under the curve (AUC) values in H. pylori‐positive patients in Study I. Receiver operating characteristic (ROC) curves. The AUC values were 0.595 for visibility of vascular pattern —, 0.564 for not having regular arrangement of collecting venules (RAC) in angle —, 0.566 for mucus —, Reference line —. [file DEO2-5-e70029-s001.jpg]
